# Submarine groundwater discharge derived strontium from the Bengal Basin traced in Bay of Bengal water samples

**DOI:** 10.1038/s41598-018-22299-5

**Published:** 2018-03-12

**Authors:** Ramananda Chakrabarti, Surajit Mondal, Shiba Shankar Acharya, J. Sree Lekha, Debasis Sengupta

**Affiliations:** 1 Centre for Earth Sciences, Indian Institute of Science, Bangalore, 560012 India; 2 Interdisciplinary Centre for Water Research, Indian Institute of Science, Bangalore, 560012 India; 3 Centre for Atmospheric and Oceanic Science, Indian Institute of Science, Bangalore, 560012 India

## Abstract

Evaluating the submarine groundwater discharge (SGD) derived strontium (Sr) flux from the Bengal Basin to the Bay of Bengal (BoB) and determining its isotopic composition is crucial for understanding the marine Sr isotopic evolution over time. Measurements of spatially and temporally distributed water samples collected from the BoB show radiogenic ^87^Sr/^86^Sr, high Sr, calcium (Ca) concentrations and high salinity in samples collected dominantly from 100–120 m depth, which can be explained only by the contribution of saline groundwater from the Bengal Basin. These results provide a direct evidence of the SGD-Sr flux to the BoB. This SGD-Sr flux is however, spatially heterogeneous and using conservative hydrological estimates of the SGD flux to the BoB, we suggest a SGD Sr flux of 13.5–40.5 × 10^5^ mol/yr to the BoB. Mass balance calculations using Sr concentrations and ^87^Sr/^86^Sr suggest up to 7% contribution of SGD to the 100–120 m BoB water samples. The identification of SGD at 100–120 m depth also provides an explanation for the anomalous variations in barium (Ba) concentrations and the δ^18^O-salinity relationship in intermediate depths of the BoB.

## Introduction

The Bay of Bengal (BoB) receives large fluxes of continental Sr, characterized by radiogenic Sr isotopic (^87^Sr/^86^Sr) composition, from the Himalayan mountain belt. Strontium is carried, dominantly as part of the dissolved load, by large rivers like the Ganges, Brahmaputra as well as the Irrawaddy, to the BoB. The ^87^Sr/^86^Sr ratios of the Himalayan outflows have played a major role in modulating the global marine Sr budget^1–4^ and the rise of the Himalayas has led to a marked increase in the ^87^Sr/^86^Sr ratio of the average global seawater over the last 40 Ma^5^. In addition to the surface flows, submarine groundwater discharge (SGD) was proposed as a significant source of continental Sr to the BoB^6^. While the seasonal and spatial variations in Sr concentrations and ^87^Sr/^86^Sr ratios of the large Himalayan rivers have been well-studied^7–10^, those of the subsurface flow are poorly constrained and their role in modulating the marine ^87^Sr/^86^Sr is debated due to contrasting observations, as described below.

**Figure 1:**
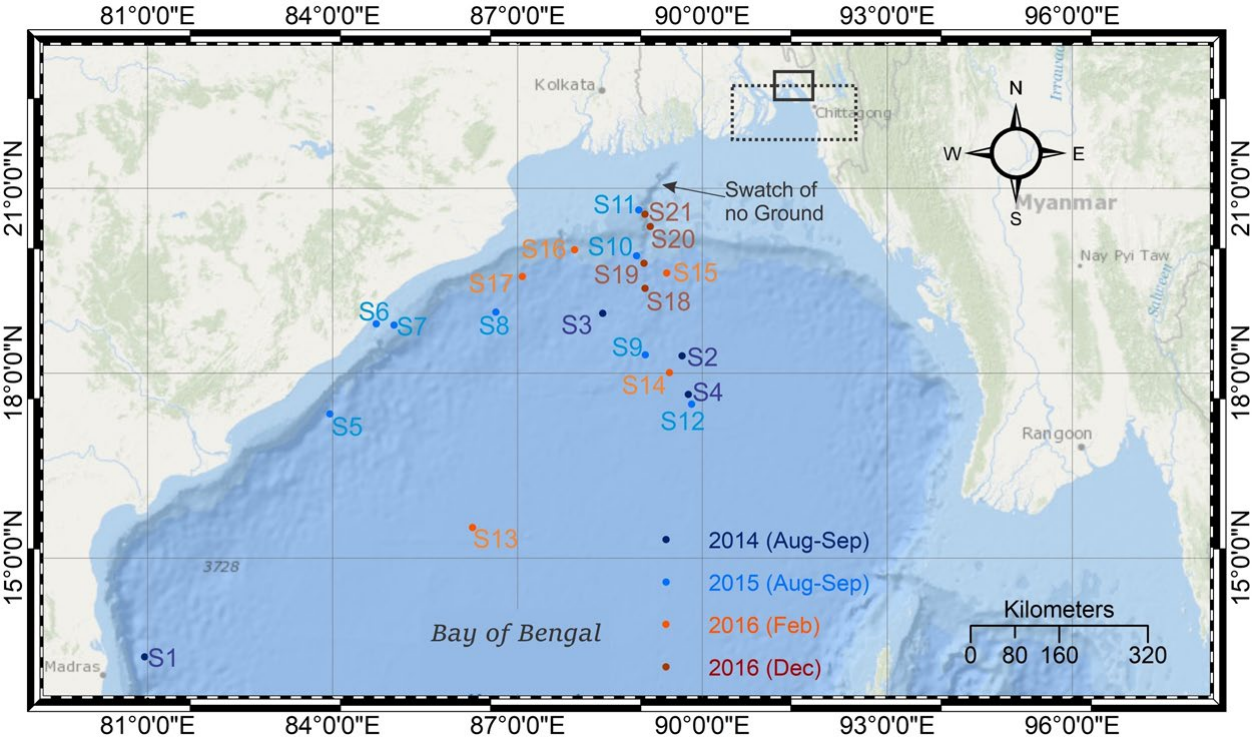
Locations of the water sampling sites of the present study in the Bay of Bengal (BoB) along with the broad study region of Moore^11^, Carroll *et al*.^12^ (dotted-rectangle) and Beck *et al*.^17^ (solid-rectangle). The samples collected in August/September 2014 (dark blue) and August/September 2015 (light blue) were during the summer monsoon when both river discharge and rainfall are high while samples collected in February 2016 (orange) and December 2016 (red) were during the dry winter months (non-monsoon) when river discharge is low^18^. The figure was prepared using the ArcMAP application in the ArcGIS software (v. 10.3) and CorelDraw X5.

The contribution of the SGD to the BoB was proposed to explain the high Ba concentrations (~460–740 nM) in surface waters of north-eastern coastal BoB (Fig. 1) sampled during the dry season (March), when the monsoon-driven riverine discharge and associated particle load is low^11^. An alternate explanation for this high Ba concentration in surface waters of BoB during the dry season is the desorption of Ba from older sediments due to the interaction with saline water^12^. Since Ba is a non-conservative tracer^13^, it is difficult to ascertain whether the high Ba concentrations in the surface BoB water samples during the dry months are source dependent (SGD versus riverine) or process dependent (e.g., adsorption and/or desorption of older particulate matter). In contrast to Ba, Sr is relatively well-mixed in the oceans and is a conservative tracer^5^. Groundwater samples of the Bengal Basin show high Sr concentration (average 4.5 μmol/l) which is an order of magnitude higher than that in the Himalayan rivers^6^. Based on these high Sr concentrations as well as hydrological considerations (high recharge rates of ~60–80 cm/yr when normalized by the land area and a high flux of SGD of 2 × 10^11^ m^3^/yr), it was proposed that the subsurface flowing groundwaters of the Bengal Basin contribute an equal amount of Sr to the BoB as the Ganges-Brahmaputra rivers^6,14^. However, this high SGD flux value^6^ was questioned^15^ and it was argued that this value could be biased by groundwater pumping in the Bengal Basin. Using regional hydrological modelling, the recharge rate was estimated to be 0.4 cm/yr, when normalized by a regional land area of 2.5 × 10^11^ m^2^, and the SGD flux from the Bengal Basin was estimated to be 3 × 10^8^ m^3^/year with post-development pumping taken into account and up to 9 × 10^8^ m^3^/year without pumping^16^ which was two to three orders of magnitude lower than the original proposal^6^. Additionally, the radiogenic ^87^Sr/^86^Sr ratio of the groundwater (0.7150–0.7200)^6^ was not detected in measurements of surface water samples of the BoB^17^ collected during the dry months of January and February from the north-eastern BoB (Fig. 1), thereby questioning the role of the Bengal Basin SGD in modulating the marine Sr isotopic composition.

### Geochemical and Sr isotopic compositional heterogeneity in Bay of Bengal water samples

Seawater samples were collected from 21 locations in the northwestern BoB, west of the sampling locations of earlier studies^11,12,17^ (Fig. 1). These samples include surface water as well as sub-surface water and were collected over a time span of 29 months from August 2014 to December 2016 from the open ocean (Fig. 1, Table 1). The samples collected in 2014 (August/September) and 2015 (August/September) were during the summer monsoon season when both river discharge and rainfall are high while samples collected in February 2016 and December 2016 were during the dry winter months (non-monsoon) when river discharge is low^18^. Strontium and Ca concentrations in the water samples were measured using an inductively coupled plasma mass spectrometer (ICPMS, Thermo Scientific X-Series II), while Sr isotope ratio measurements (^87^Sr/^86^Sr) were performed using a thermal ionization mass spectrometer (TIMS, Thermo Scientific Triton Plus), both at the Centre for Earth Sciences (CEaS), Indian Institute of Science (IISc). Salinity of the water samples were measured *in-situ*. See Methods for additional details.

**Table 1:** Geochemical and Sr isotopic compositions of the Bay of Bengal water samples along with their date of sampling, sampling locations and depth of sampling.

Sample location	Sampling depth (m)	Date of sampling	Latitude	Longitude	salinity (psu)	Ca (mmol/l)	Sr (μmol/l)	^87^Sr/^86^Sr	Δ^87^Sr/^86^Sr (ppm)	Ca/Sr (μmol/μmol)	1000 x Salinity /Sr (psu/μmol/l)
S1	0	2014_Aug23	13°24.139′N	80°56.89′E	32.700	9.58	79.39	0.709148	−32	120.7	412
″	100	″	″	″	34.754	10.44	87.82	0.709188	24	118.9	396
S2	0	2014_Aug28	18°17.162′N	89°40.215′E	30.580	9.32	76.22	0.709158	−18	122.3	401
″	100	″	″	″	34.645	10.08	85.92	0.709285	161	117.3	403
S3	0	2014_Aug29	18°58.56′N	88°22.95′E	28.501	9.11	73.99	0.709213	59	123.1	385
S4	0	2014_Sep03	17°39.60′N	89°46.232′E	31.097	9.35	76.41	0.709166	−7	122.4	407
″	100	″	″	″	34.759	10.65	88.36	0.709262	128	120.6	393
S5	0	2015_Aug24	17°20.692′N	83°57.34′E	30.405	6.26	51.23	0.709192	30	122.3	593
S6	0	2015_Aug25	18°48.24′N	84°42.28′E	32.708	10.11	83.09	0.709162	−13	121.6	394
S7	0	2015_Aug25	18°47.24′N	84°59.65′E	33.126	9.74	79.46	0.709204	47	122.5	417
″	100	″	″	″	34.709	10.10	85.27	0.709172	1	118.4	407
S8	0	2015_Aug26	18°59.806′N	86°38.806′E	32.190	9.86	80.08	0.709216	63	123.1	402
S9	0	2015_Aug28	18°18.114′N	89°4.335′E	31.460	8.71	69.61	0.709194	32	125.1	452
S10	0	2015_Aug29	19°54.515′N	88°55.826′E	33.262	10.80	83.84	0.709218	66	128.8	397
″	100	″	“	″	34.853	10.47	86.83	0.709270	140	120.5	401
S11	0	2015_Aug30	20°39.285′N	88°57.911′E	29.890	8.80	72.27	0.709189	25	121.8	414
S12	0	2015_Sep01	17°30.019′N	89°49.358′E	30.338	9.27	74.42	0.709187	23	124.5	408
″	100	″	″	″	34.433	9.96	85.42	0.709195	34	116.6	403
S13	0	2016_Feb01	15°29.95′N	86°16.21′E	32.943	9.75	80.05	0.709191	28	121.8	412
″	100	″	″	″	33.250	10.46	87.21	0.709193	31	120.0	381
S14	0	2016_Feb03	18°00.77′N	89°27.98′E	30.924	9.39	76.67	0.709185	20	122.4	403
″	100	″	″	″	34.770	10.56	87.71	0.709217	65	120.4	396
S15	0	2016_Feb05	19°37.67′N	89°25.20′E	31.674	9.56	77.23	0.709175	6	123.8	410
″	100	″	″	″	34.578	10.47	87.24	0.709172	1	120.0	396
S16	0	2016_Feb07	20°00.34′N	87°55.64′E	31.048	9.19	75.48	0.709151	−28	121.8	411
″	100	″	″	″	34.761	10.24	86.91	0.709196	35	117.8	400
S17	0	2016_Feb09	19°34.38′N	87°04.72′E	29.704	8.93	73.09	0.709197	37	122.1	406
″	100	″	″	″	34.826	10.50	88.04	0.709339	237	119.2	396
S18	0	2016_Dec20	19°22.98′N	89°04.02′E	30.746	9.33	77.03	0.709170	−1	121.1	399
″	50	″	″	″	32.569	9.84	82.22	0.709163	−11	119.7	396
″	120	″	″	″	34.788	10.46	88.66	0.709167	−6	118.0	392
″	350	″	″	″	35.029	10.53	89.76	0.709176	7	117.3	390
″	700	″	″	″	35.001	10.71	89.62	0.709170	−1	119.5	391
″	1000	″	″	″	34.942	10.57	89.04	0.709173	3	118.7	392
″	1500	″	″	″	34.844	10.53	88.70	0.709175	6	118.8	393
S19	0	2016_Dec21	19°47.22′N	89°03.06′E	32.514	9.86	81.54	0.709166	−7	121.0	399
″	50	″	″	″	32.639	10.54	86.93	0.709168	−4	121.3	375
″	120	″	″	″	34.820	10.57	89.02	0.709166	−7	118.7	391
″	350	″	″	″	35.035	10.88	90.36	0.709177	8	120.4	388
″	700	″	″	″	34.998	10.71	90.02	0.709168	−4	118.9	389
″	1000	″	″	″	34.936	10.94	91.32	0.709170	−1	119.8	383
″	1500	″	″	″	34.830	10.33	87.07	0.709172	1	118.6	400
S20	0	2016_Dec22	20°34.98′N	89°4.02′E	32.195	9.56	80.00	0.709189	25	119.5	402
″	50	″	″	″	32.477	9.47	79.08	0.709176	7	119.8	411
″	120	″	″	″	34.645	9.95	84.86	0.709168	−4	117.3	408
″	250	″	″	″	35.028	10.10	85.99	0.709165	−8	117.4	407
″	450	″	″	″	35.026	10.15	86.97	0.709162	−13	116.7	403
″	750	″	″	″	34.969	10.51	87.82	0.709172	1	119.7	398
″	1000	″	″	″	34.921	10.50	88.09	0.709169	−3	119.2	396
S21	0	2016_Dec23	20°23.04′N	89°9.06′E	32.303	9.45	79.12	0.709170	−1	119.4	408
″	50	″	″	″	32.765	9.61	80.28	0.709167	−6	119.6	408
″	120	″	″	″	34.693	10.36	87.51	0.709175	6	118.4	396
″	250	″	″	″	35.022	10.47	87.12	0.709163	−11	120.1	402
″	450	″	″	″	35.012	10.09	86.76	0.709173	3	116.3	404
″	700	″	″	″	34.966	10.30	87.96	0.709170	−1	117.1	398
″	1000	″	″	″	34.919	10.28	87.31	0.709176	7	117.8	400
Average of 0–50 m samples					31.630 ± 2.498			0.709181 ± 39			
Average of 100–120 m samples					34.619 ± 0.788			0.709211 ± 106			
Average of D-BoB samples (>250 m)					34.967 ± 0.129			0.709171 ± 9			

Δ^87^Sr/^86^Sr is defined as [(^87^Sr/^86^Sr _(sample)_/0.709171-1) × 10^6^], where 0.709171 is the average ^87^Sr/^86^Sr of deep BoB (D-BoB) water samples collected from >250 m depth which overlaps with the average ^87^Sr/^86^Sr of global seawater^21^. Concentrations of Ca and Sr are measured in ppm using ICPMS. Calcium (ppm) is converted to mmol/l by multiplying 1/39.926 while Sr (ppm) is converted to μmol/l by multiplying 1000/87.9056.

Due to its conservative nature, ^87^Sr/^86^Sr ratio of the open ocean is broadly homogeneous. The homegeneity in seawater ^87^Sr/^86^Sr has been established by direct measurements of deep and shallow water samples from the Hudson Bay and the Pacific, Atlantic, Indian and Arctic oceans^19–21^ as well as by measurements of biogenic carbonates^22^ and marine barite from Holocene sediments from the Pacific, Atlantic and Indian oceans^23^. The maximum variability in the ^87^Sr/^86^Sr ratio of seawater at any given time is expected to be less than 10 ppm^19,20^. In contrast, significant variations in the ^87^Sr/^86^Sr ratio are observed in the water samples of the BoB collected from different depths. Samples collected from depths greater than 250 m show limited variability and the average ^87^Sr/^86^Sr ratio of these samples (0.709171 ± 9, 2 SD, n = 16) (Table 1) overlaps with that of the average global seawater (0.709179 ± 8, 2 SD)^21^. Samples collected from 0–50 m depth show a larger range in ^87^Sr/^86^Sr (0.709148–0.709218, average = 0.709181 + 39, 2 SD, n = 25). Significant variations are observed in ^87^Sr/^86^Sr ratios (0.709166–0.709339, average = 0.709211 ± 106, 2 SD, n = 15) of the samples collected from 100–120 m depth (Table 1). Salinity of the BoB water samples, consistent with previous depth-profile measurements^24^, also show significant variability (28.501–35.035 psu). The average salinity increases while the 2 SD of the average value decreases in samples collected from 0–50 m (31.630 ± 2.498 psu) to 100–120 m (34.619 ± 0.788 psu) to >250 m (34.967 ± 0.129 psu). The deep BoB water samples show salinity values that overlap with global oceans (~35 psu)^25^.

**Figure 2:**
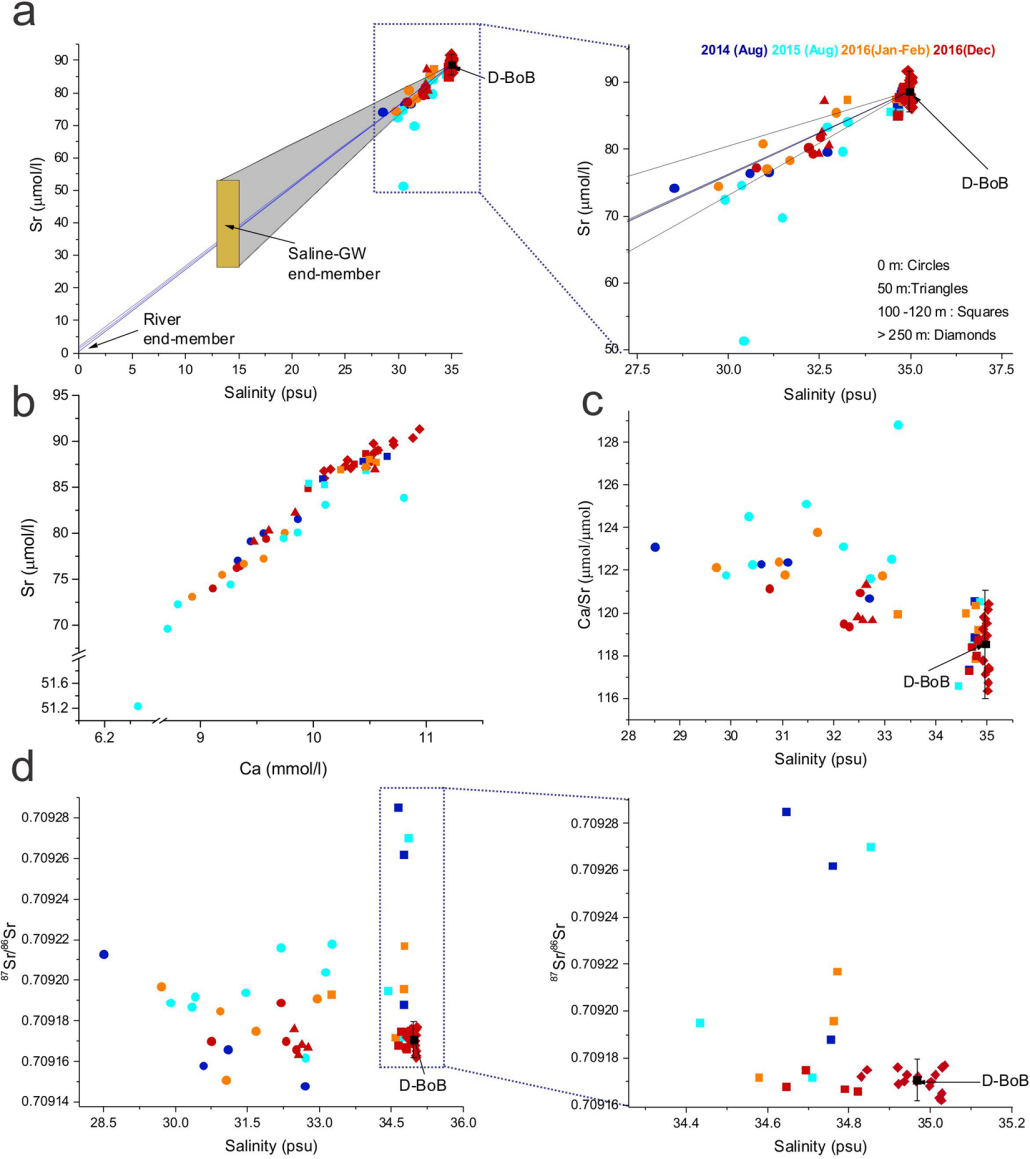
Variations in salinity, Sr and Ca concentrations, Ca/Sr and ^87^Sr/^86^Sr ratios of the BoB water samples of this study. (**a**) A plot of Sr concentration versus salinity where the samples collected from 0 m (circles) and 50 m (triangle) mostly plot on mixing lines (blue) between the compositions of the average deep BoB water (D-BoB, >250 m) and the Ganges, Brahmaputra and Irrawaddy rivers. Also shown for comparison is the composition range of saline groundwater from the Bengal Basin (end-members compositions are reported in Table 2). (**b**) A plot of Ca and Sr concentrations in the water samples show co-variation with the samples collected from 0 m and 50 m showing relatively low concentrations. (**c**) A plot of Ca/Sr versus salinity where samples collected from 0–50 m clearly show low salinity and high Ca/Sr compared to the deeper water samples. (**d**) A plot of ^87^Sr/^86^Sr versus salinity where D-BoB samples show constricted values overlapping with the composition of average global seawater^21^. Samples from 0–50 m and 100–120 m show some variation in ^87^Sr/^86^Sr with the most radiogenic ^87^Sr/^86^Sr observed in samples collected from 100–120 m depths.

**Figure 3:**
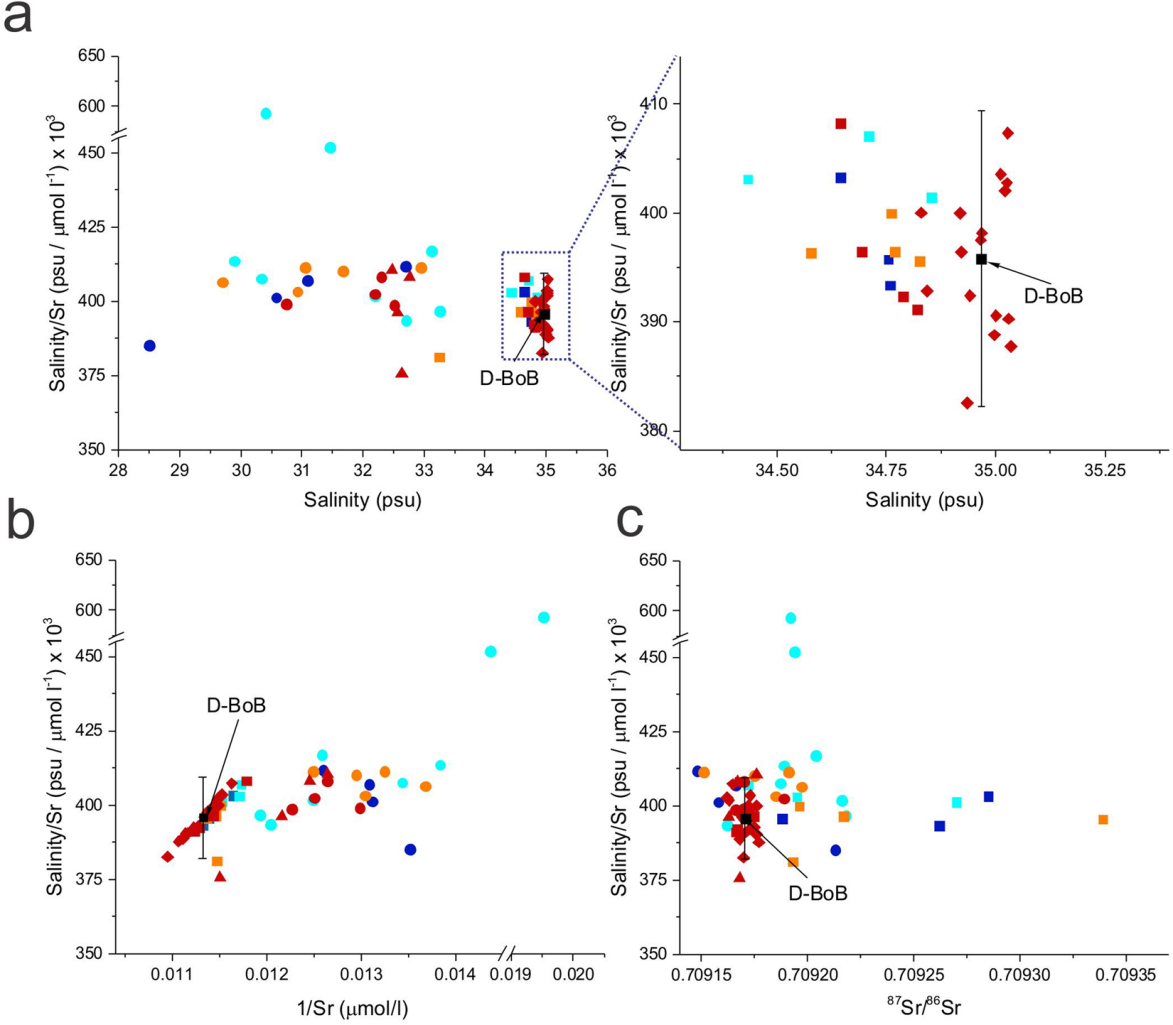
Plots of **(a)** salinity/Sr versus salinity, (**b**) salinity/Sr versus 1/Sr and (**c**) salinity/Sr versus ^87^Sr/^86^Sr. (**a**) Barring two 0 m samples collected during monsoon, all BoB water samples show similar salinity/Sr values which overlap with global seawater values^27^ (**b**) The 0–50 m water samples from the BoB are clearly distinguished from the deeper BoB water samples in a plot of Salinity/Sr versus 1/Sr. While the deeper water samples show a positive correlation, the 0–50 m samples do not show any correlation and show distinctly higher 1/Sr values (**c**) In a plot of salinity/Sr versus ^87^Sr/^86^Sr, water samples from 100–120 m depth are clearly distinguished from the other deeper water samples by their radiogenic ^87^Sr/^86^Sr.

Concentrations of Sr (51.23–91.32 μmol/l) and Ca (6.26–10.94 mmol/l) show significant variability in the 56 BoB water samples with samples collected from 0 m and 50 m depth showing significantly lower Sr and Ca (Fig. 2, Table 1). Strontium concentrations in global seawater show variations (e.g., 7.40–8.79 ppm^26^ or 84.18–99.90 μmol/l). Within the Pacific Ocean, Sr concentrations range from 7.21 ppm to 8.43 ppm which is equivalent to 82.02–95.90 μmol/l^25–27^. Globally, surface seawater samples typically show lower Sr concentrations compared to deeper water^25^ while near shore samples also show relatively low values^26^. The Sr concentrations in the BoB water samples overlap with that reported for global oceans^25–27^. However, the slightly lower concentrations, especially in the 0–50 m depth samples of the BoB, could be due to large inputs from the Ganges, Brahmaputra and Irrawaddy rivers which have very low Sr concentrations (Table 2) as well as dilution due to rainwater contribution. It has been suggested that the salinity/Sr value of ocean water is constant^27^ although, some studies suggest otherwise^28^. For the BoB water samples, the salinity/Sr values for the surface water samples overlap with those of the deep-water samples (Fig. 3, Table 1). A couple of surface water samples (locations S5, S9) collected during the monsoon show high salinity/Sr due to the dilution effect of rainwater which has very low Sr^29^. The concentrations of Ca and Sr in the BoB water samples show a positive correlation (R^2^ = 0.95, Fig. 2b) which is consistent with the similar behavior of these two elements in aqueous systems. However, the Ca/Sr ratio of the samples collected from 0 m and 50 m show mostly higher values compared to the deeper water samples (Fig. 2c).

**Table 2:** Geochemical and Sr isotopic compositions of the Ganges, Brahmaputra and Irrawaddy rivers, saline groundwater from the Bengal Basin and average global seawater.

End-member	Data reference	Salinity	Sr (μmol/l)	1/Sr	^87^Sr/^86^Sr
Ganges River	Basu *et al*.^6^ (Fig. 2, Table 1)	0.02*	2	0.50	0.7249
	(samples with the highest and lowest Sr)	0.02*	0.55	1.82	0.7243
Brahmaputra River	Basu *et al*.^6^ (Fig. 2, Table 1)	0.02*	1.17	0.85	0.726
	(samples with the highest and lowest Sr)	0.02*	0.55	1.82	0.7171
Irrawaddy River	Chapman *et al*.^10^	0.02*	1.36	0.74	0.717635
	(samples with the highest and lowest Sr)	0.02*	0.58	1.72	0.709519
Saline Groundwater	Dowling *et al*.^14^	15	26.55	0.04	0.710301
	(samples BGD22, BGD23)	13	52.74	0.02	0.714327
Av. Global Seawater	Mokadem *et al*.^21^				0.709179 ± 0.000008 (2 SD)
	Richter *et al*.^5^, Basu *et al*.^6^		91.3		
	De Villiers^25^	35			

^*^Salinity values for river water (Ganges, Brahmaputra and Irrawaddy) were not reported in the above references and are assumed to be 0.02 psu.

The variations in the geochemical and Sr isotopic compositions in the BoB water samples could be due to multiple reasons that include: (i) local hydrothermal input and/or oceanic crust - seawater interaction at mid-oceanic ridges^30^, (ii) differential atmospheric contributions (aerosol and/or rainwater)^31^, (iii) biomineralization and its subsequent dissolution^25^, (iv) dissolution of riverine particulate matter^32^ and (v) mixing of different water masses^33^. The absence of any active mid-oceanic ridge in the BoB rules out any local hydrothermal inputs. The atmospheric contribution of Sr to the Bengal basin was estimated to be 21 nmol/l^8^ while Sr concentrations in rainwater were estimated to be 32–191 nM^29^. These concentrations are very low to affect the oceanic Sr reservoir with an average Sr concentration of 91.3 μmol/l^5^. In addition, the lack of any variations in Sr, Ca concentrations, salinity and as well as ^87^Sr/^86^Sr ratios between samples collected during the monsoon (2014, 2015) and non-monsoon (2016) (Fig. 2), all indicate that atmospheric contributions could not have affected the ^87^Sr/^86^Sr of the BoB water samples. Biomineralization of Celestite (SrSO_4_), tests of the protozoan acantharia and its dissolution^25,34^ have been invoked to explain the ~5% variation^25^ in the Sr concentrations with depth in certain oceanic basins. However, these organisms have not been reported in the BoB. In addition, the lack of seasonal variation in the Sr, Ca concentrations and ^87^Sr/^86^Sr ratios in the monsoon-driven biogeochemical system of BoB, (Fig. 2) rules out the possibility of any biogenic contribution to the Sr isotopic variation of BoB. The dissolution of riverine particulate matter can also change the ^87^Sr/^86^Sr ratio of seawater samples^32,35^. If the dissolution of particulate sediments is the source of the high ^87^Sr/^86^Sr in the BoB water samples, a seasonal variation would be expected in the Sr concentration and ^87^Sr/^86^Sr of the BoB water as the suspended sediment load in the rivers is very high during the monsoon. In addition, the effect of sediment dissolution is expected to be higher in the samples collected from closer to the coast where the riverine influence is higher compared to the samples collected from father away. However, the radiogenic ^87^Sr/^86^Sr ratios observed in the BoB water samples, particularly in the samples collected from 100 m depth, which were collected both during the monsoon and non-monsoon seasons do not show any seasonal variations as well as any relationship with the sampling distance from the coast (Figs 1, 2). Therefore, the contribution from the dissolution of particulate matter can be ruled out. Hence, the observed variations in the Sr concentrations and ^87^Sr/^86^Sr as well as salinity in the BoB samples (Figs 2, 3) can only be explained by the mixing of water from different sources.

### Contributions of river water and evidence for submarine groundwater discharge

The samples of this study can be divided into three distinct groups based on their Sr, Ca concentration, salinity, and ^87^Sr/^86^Sr (Figs 2, 3). The first group comprises samples collected from 0–50 m depths which show lower salinity, Sr and Ca concentrations and higher Ca/Sr compared to the other samples. Strontium concentrations, especially in the samples collected from the surface, show a rough positive correlation with salinity and fall on a mixing trend between the average composition of the deep waters of BoB (>250 m, D-BoB) (Fig. 2a), which overlaps with the composition of the average global seawater, and the average compositions of the Ganges, Brahmaputra and Irrawaddy rivers (Table 2).

**Figure 4:**
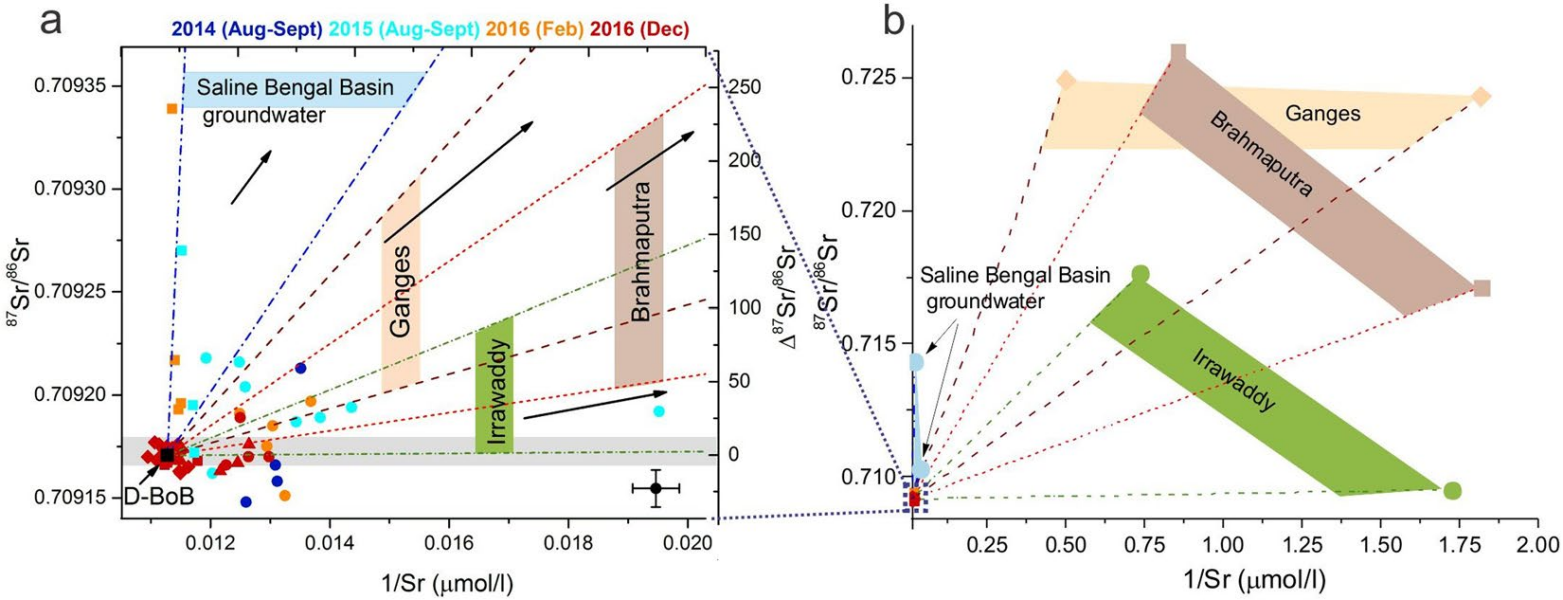
A plot of ^87^Sr/^86^Sr versus 1/Sr showing: (**a**) compositional variations of the BoB water samples and (**b**) those of the Ganges, Brahmaputra and Irrawaddy rivers and saline groundwater from the Bengal Basin (Tables 1, 2). The samples from 0–50 m and 100–120 m show significant variations in ^87^Sr/^86^Sr ratios, which are greater than our analytical uncertainty (±9 ppm) (gray shade and cross-hairs). The compositional variations in the BoB samples from 0–50 m can be explained by mixing between the average D-BoB and the Ganges^6^, Brahmaputra^6^ and Irrawaddy^10^ rivers. Compositions of several samples from 100–120 m depth (squares) with very radiogenic ^87^Sr/^86^Sr and low 1/Sr (2 SD, ±0.0004, shown as cross-hairs) require contributions from saline groundwater from the Bengal Basin^14^.

These samples also show large variations in ^87^Sr/^86^Sr, with mostly radiogenic values compared to the average D-BoB which overlaps with the composition of the global average seawater^21^ (Table 2) (Figs 2b, 4). In a plot of ^87^Sr/^86^Sr versus 1/Sr, the composition of these samples can be explained by mixing between the average D-BoB and river water end-members comprising Ganges, Brahmaputra, and Irrawaddy (Fig. 4). The compositional uniqueness of the 0–50 m samples is also evident in a plot of salinity/Sr versus 1/Sr (Fig. 3b) where, unlike the deeper water samples, these samples show large variations in Sr concentrations and no correlation between salinity/Sr and 1/Sr. The low salinity of these samples suggests the dominance of the riverine input although, the influence of groundwater cannot be ruled out. The exchange of shallow groundwater (<40 m) with the Ganges-Brahmaputra rivers has been observed in the Bengal basin, where the Sr and Li composition of both end-members overlap with each other^14,36^. The influence of groundwater is consistent with the composition of some of these samples that plot within the mixing domain of high saline groundwater and average seawater (Figs 2a and 4).

The second group comprises samples collected mostly from the deeper waters (>250 m depth, D-BoB) that are characterized by limited variations in Sr concentrations, average global seawater-like ^87^Sr/^86^Sr ratios, high values of salinity tightly clustered around 34.967 ± 0.129 psu (2 SD) and a strong correlation between salinity/Sr and 1/Sr (Fig. 3b). The Sr concentrations of these samples overlap with those of the Pacific and Atlantic oceans as reported by Bernat *et al*.^27^ and De Villiers^25^ but are lower than that reported by Wakaki *et al*.^26^.

The third group consists of samples that are collected from 100–120 m depth and are characterized by significantly radiogenic ^87^Sr/^86^Sr ratios compared to D-BoB (average ^87^Sr/^86^Sr = 0.709171), as high as 0.709339, but unlike the 0–50 m samples, these have markedly higher salinity and Sr, Ca concentrations (Table 1, Fig. 2). The salinity of these samples is however, slightly lower than the >250 m samples (Table 1, Figs 2d and 3a). Eight out of 14 samples from 100–120 m depth show more radiogenic ^87^Sr/^86^Sr ratios than that of the D-BoB. This excess in ^87^Sr/^86^Sr is expressed as Δ^87^Sr/^86^Sr (defined as [^87^Sr/^86^Sr _(sample)_/0.709171-1] × 10^6^) and ranges from 24 ppm to as high as 237ppm (Fig. 4). We consider samples whose Δ^87^Sr/^86^Sr values are higher than the analytical uncertainty of the measurements (9 ppm, 2 SD) based on the external reproducibility of the average D-BoB (n = 16) (Table 1). The BoB is highly stratified with a strong shallow pycnocline at 10–30 m depth that inhibits mixing between surface and sub-surface water^37^. Rare exceptions can occur during tropical cyclones. For example, water characteristic of the Andaman Sea was observed within the thermocline of the western Bay of Bengal during the passage of the Tropical Cyclone Lehar^38^. However, the relatively high salinity of the water samples from 100–120 m depth (33.250–34.853) compared to the water samples from 0–50 m depth (28.501–33.262) (Table 1) and the lack of seasonal variability in their ^87^Sr/^86^Sr ratios, which is expected due to the varying fluxes of the monsoon-driven rivers draining into the BoB, both rule out the possibility of a riverine water input at 100–120 m depth. Therefore, we propose that the high Δ^87^Sr/^86^Sr of the water samples collected from the 100–120 m depths are a result of radiogenic Sr input of SGD from the Bengal Basin. The Sr input from a SGD is further corroborated in a plot of ^87^Sr/^86^Sr versus 1/Sr (Fig. 4), where compositions of the high Δ^87^Sr/^86^Sr water samples can only be explained by the mixing between average seawater and saline groundwater^6,14^ from the Bengal Basin (Table 2). This observation is also in-line with the results of regional groundwater modeling for the Bengal Basin which indicates the presence of large-scale groundwater flow at depths of 100 m or greater^16^.

### Submarine groundwater discharge and Ba, δ^18^O profiles

High concentrations of the non-conservative tracer Ba in suraface waters of BoB during non-monsoon was interpreted as a signature of SGD from the Bengal Basin^11^. In depth profiles of Ba concentrations in the BoB^39^, dissolved Ba in the coastal and open waters of the BoB show depleted concentrations in the surface and enrichments in the deeper layers, a pattern similar to other oceanic systems^40^. Compared to the north-eastern BoB^11^ (Fig. 1), the Ba concentrations in the surface water samples of western BoB were found to be lower^39^. However, an increase in the Ba concentration below the shallow mixed layer (~60–120 m depth) was observed in most of the profiles^39^. One possible explanation for the increase in Ba concentrations at 60–120 m depths in northern BoB^39^ is the desorption of Ba from particulate matter supplied by the SGD. However, without detailed characterization of the nature of the SGD to the BoB, the above explanation is speculative. At the same water depths, the correlation between δ^18^O and salinity in the BoB was found to be poor^24^, which was explained by the mixing of two distinct water masses having very different δ^18^O values. One of these two water masses were suggested as Indonesian Throughflow (ITF) while the second water mass was unidentified^24^. This unidentified water mass at intermediate depths in the BoB could be the Bengal Basin SGD, as identified in the present study.

### Spatial heterogeneity in the submarine groundwater discharge

The high concentration of Sr in the Bengal Basin groundwater and its radiogenic ^87^Sr/^86^Sr composition led to the suggestion that SGD is an important source of continental Sr to the oceans and that it carries an equal in magnitude of dissolved Sr as the Ganges-Brahmaputra rivers^6^. The importance of constraining the submarine groundwater Sr flux from the Bengal Basin for evaluating the global marine Sr budget was recognized but signatures of the SGD with radiogenic ^87^Sr/^86^Sr were not found in measurements of the surface waters of BoB collected during non-monsoon (January-February) near Bangladesh^17^ (Fig. 1). It may be noted that Beck *et al*.^17^ did not analyze any deeper water samples. In the present study, the radiogenic Sr isotopic signature of the SGD is observed in the intermediate water layers (100–120 m depth) in the north-western BoB, off the coast of India. However, the radiogenic ^87^Sr/^86^Sr signature is not observed in all the samples collected from these depths. Additionally, while the SGD signature is observed in samples away from the coast, it was not detected in four depth profiles along a traverse from the Swatch of No Ground (locations S18-S21) (Fig. 1), where the Sr-isotopic composition is very similar to the D-BoB (Table 1). The above observations suggests a spatially heterogeneous nature of the SGD flow in BoB. In the BoB, spatial heterogeneity is observed in the flow of river water in the surface BoB which is stirred by mesoscale eddies, often creating filament-like structures of low salinity waters and sharp fronts between freshwater and relatively saltier water^41^. A heterogeneous flow pattern of SGD from the Bengal Basin is consistent with spatially heterogeneous flow pattern of coastal SGD observed in Ubatuba, Brazil^42^ and could explain the contrasting findings of the present study and that of Beck *et al*.^17^.

### Sr flux to the BoB from submarine groundwater discharge

The identification of the SGD signature with radiogenic ^87^Sr/^86^Sr, high Sr and high salinity at intermediate water depths of the BoB is consistent with the suggestion of Basu *et al*.^6^ regarding Sr inputs from the SGD to the BoB. However, given that the Sr-flux of SGD to the BoB from the Bengal Basin is heterogeneous, the SGD flux estimates to the BoB by Basu *et al*.^6^ were possibly overestimates, as also suggested by regional hydrological modelling studies^16^. Using the Bengal Basin groundwater fluxes of Michael and Voss^16^ which range from 3 × 10^8^ m^3^/yr (post-groundwater pumping) to 9 × 10^8^ m^3^/yr (pre-groundwater pumping) and the average Sr concentration of Bengal Basin groundwater of 4.5 μmol/l^6^, the SGD driven Sr flux to the BoB is estimated between 13.5 × 10^5^ mol/yr and 40.5 × 10^5^ mol/yr, which is three orders of magnitude lower than the estimates of Basu *et al*.^6^ and Dowling *et al*.^14^. Two-component mixing calculations, using Sr concentrations and ^87^Sr/^86^Sr compositions, between a saline Bengal Basin groundwater end-member (BGD22, 23)^14^ and the average deep BoB end-member (Tables 1, 2) (Fig. 4) suggests that for the samples with high Δ^87^Sr/^86^Sr in the BoB, the contribution of Sr from the saline Bengal Basin groundwater end-member is as high as 7%. Similar mass balance calculations using Sr concentrations and ^87^Sr/^86^Sr compositions of the Ganges, Brahmaputra or Irrawaddy rivers and those for the D-BoB end-member (Tables 1, 2) suggest that the contribution of river water to the surface water composition at BoB can range from 8–21%. The high Sr contribution from the Bengal Basin groundwater (~7%), although, spatially restricted due to the heterogeneous nature of the SGD, is higher than estimates for Li contribution from the Bengal basin groundwater (~2%) to the BoB^36^ and could have implications for estimates of continental Sr input to the oceans back in time.

## Methods

Seawater samples from 21 locations in the northwestern BoB were collected during four different sampling missions aboard the ORV Sagar Kanya and ORV Sagar Nidhi over a time span of 29 months between August 2014 and December 2016 (Fig. 1, Table 1). The samples were collected from the open ocean. Sub-surface seawater samples were collected using 5 L Niskin bottles, mounted on a 12-bottle rosette with a Seabird CTD. The surface water samples were collected using a plastic bucket while the ship was stationed for CTD measurements. Water sampling at greater depths (depth-profiles) was not possible at all sampling locations. All samples were acidified with double-distilled nitric acid (pH ~2) immediately after sampling. The salinity data was measured *in-situ* from CTD (SeaBird SBE 19 Plus CTD system).

The concentrations of Sr and Ca were measured using an Inductively Coupled Plasma Mass Spectrometer (ICPMS, Thermo Scientific X-Series II) at the Centre for Earth Sciences (CEaS), Indian Institute of Science (IISc). For these measurements, ultra-pure ICPMS solutions from Alfa Aesar (traceable to NIST standards) were used as calibrations standards (four-point calibration). A 10 ppb Be, In solution was used as an internal standard to correct instrumental drift. External reproducibility (2 SD) of the Sr (±2.33) and Ca (±0.47) concentrations and 1/Sr (±0.0004) were estimated based on multiple measurements of a seawater standard NASS 6 (n = 46) measured in two different analytical sessions along with repeat measurements of the samples. The concentrations of Sr and Ca in the deep-water samples of the BoB are consistent with the deep-water measurements of global oceans^25–27^.

For isotopic measurements, Sr was separated from the samples using cation-exchange chromatography. The purified Sr, was loaded on single Ta filaments and measured using a Thermal Ionization Mass Spectrometer (TIMS, Thermo Scientific Triton Plus) at CEaS, IISc. The NIST SRM 987 standard analyzed during the course of this study yielded an average value of 0.710268 ± 0.000022 (n = 22). The measured ^87^Sr/^86^Sr ratios were corrected for instrumental mass fractionation using ^86^Sr/^88^Sr = 0.1194. The normalized ^87^Sr/^86^Sr of the samples were re-normalized to the recommended NIST SRM 987 value of 0.710244^43^. After re-normalization, the ^87^Sr/^86^Sr ratio of the NASS 6 seawater standard, analyzed during the course of this study, yielded a value of 0.709180 ± 0.000020 (n = 4), which is consistent with the average global seawater ^87^Sr/^86^Sr value^21^ indicating that our measurements are accurate. The ^87^Sr/^86^Sr ratio of sixteen deep BoB water samples (D-BoB) collected from depths greater than 250 m yield an average value of 0.709170  ± 9 (2 SD) which is consistent with ^87^Sr/^86^Sr of global seawater value of 0.709179 ± 8^21^. The D-BoB measurements indicate that our analyses are accurate and the external reproducibility of our ^87^Sr/^86^Sr measurements is better than 9 ppm. Additional details of the elemental and isotopic measurements are described in Banerjee *et al*.^44^.
